# The diagnostic and prognostic significance of methylated arginine metabolites (ADMA, SDMA, L-NMMA) in patients with obstructive sleep apnea syndrome

**DOI:** 10.1097/MD.0000000000043903

**Published:** 2025-08-08

**Authors:** Emrah Bolca, Dilek Ergün, Recai Ergün, Fikret Kanat, Ali Ünlü, Duygu Eryavuz Onmaz, Muslu Kazim Körez

**Affiliations:** a Department of Pulmonary Medicine, Konya City Hospital, Konya, Türkiye; b Department of Pulmonary Medicine, Faculty of Medicine, Selçuk University, Konya, Türkiye; c Department of Biochemistry, Faculty of Medicine, Selçuk University, Konya, Türkiye; d Department of Biochemistry, Faculty of Medicine, Bandirma Onyedi Eylül University, Bandirma, Türkiye; e Department of Biostatistics, Faculty of Medicine, Selçuk University, Konya, Türkiye.

**Keywords:** ADMA, intermittent hypoxia, L-NMMA, NO, OSAS, SDMA, TMAL

## Abstract

In obstructive sleep apnea syndrome (OSAS), changes in the levels of methylated arginine derivatives have been observed due to intermittent hypoxia. While intermittent hypoxia initially increases the activity of the enzyme dimethylarginine dimethylaminohydrolase (DDAH), thereby reducing the levels of methylated arginine derivatives, prolonged hypoxia can disrupt this mechanism and trigger vascular damage. Therefore, methylated arginine metabolites play a critical role in the pathophysiology of OSAS. This study investigates the relationship between the L-arginine–nitric oxide (NO) pathway and methylated arginine metabolites–asymmetric dimethylarginine (ADMA), symmetric dimethylarginine (SDMA), and N-monomethyl-L-arginine (L-NMMA)–in newly diagnosed OSAS patients without comorbidities, aiming to evaluate their potential as diagnostic and prognostic biomarkers. This observational case-control study included a total of 122 participants, consisting of 31 healthy controls and 91 patients with OSAS. OSAS patients were stratified by disease severity into 30 mild, 30 moderate, and 31 severe cases. Serum levels of methylated arginine metabolites (ADMA, SDMA, and L-NMMA) and arginine were measured using mass spectrometry. The analyses were performed with an AB Sciex API 3200 triple quadrupole mass spectrometer (USA) equipped with an electrospray ionization (ESI) source operating in positive ion mode, coupled to a Shimadzu LC-20AD liquid chromatography system (Kyoto, Japan). Compared to the control group, OSAS patients showed statistically significant lower levels of ADMA, L-NMMA, arginine, and total methylated arginine load (TMAL) (*P* < .001). SDMA levels were similar across groups. In OSAS patients without comorbidities, a reduction in TMAL may suggest the activation of compensatory mechanisms in response to sleep-related intermittent hypoxia. This could reflect a shift in the arginine pathway towards enhanced nitric oxide synthesis to mitigate hypoxia-induced vasoconstriction through vasodilation. The reduced arginine levels are likely due to increased utilization, while diminished synthesis of methylated arginine derivatives (ADMA, L-NMMA) may result from this metabolic shift. These findings imply that decreased arginine and methylated arginine levels may serve as potential diagnostic markers and could aid in identifying candidates for polysomnography (PSG), which is both costly and time-consuming, thereby contributing to more efficient patient selection and reducing the overall clinical burden.

## 1. Introduction

On average, humans spend approximately one-third of their lives asleep. Obstructive sleep apnea syndrome (OSAS), which manifests during this significant portion of the day, is a major health issue classified among respiratory sleep disorders and has widespread systemic implications. OSAS is characterized by recurrent obstruction of the upper airway during sleep and is considered the most common cause of sleep-related breathing disturbances in adults.^[1,2]^

Current estimates suggest that the overall prevalence of OSAS is around 5%. Among the adult population, approximately 50% of men and 30% of women are habitual snorers, and 3 to 5% of these individuals are diagnosed with OSAS. The gold standard for the diagnosis of sleep apnea is polysomnography (PSG), a diagnostic method involving the simultaneous recording, analysis, and interpretation of multiple physiological parameters throughout sleep.^[3]^

The most frequently reported nocturnal symptom is snoring, while excessive daytime sleepiness is the predominant daytime complaint. Additional symptoms include nocturnal dyspnea, morning fatigue, restless sleep, and morning headaches. Less frequently reported manifestations include nocturia, enuresis, decreased libido, and symptoms of gastroesophageal reflux. OSAS contributes significantly to morbidity and mortality, not only through its cardiovascular consequences but also through its social and neuropsychological impacts.^[4]^

Repetitive episodes of hypoxemia and subsequent reoxygenation in OSAS patients are known to trigger oxidative stress mechanisms. Oxidative stress can alter the activity of enzymes responsible for the production of asymmetric dimethylarginine (ADMA), symmetric dimethylarginine (SDMA), and N-monomethyl-L-arginine (L-NMMA), thereby affecting their serum concentrations.^[5]^

Early intermittent hypoxia acts as a preconditioning stimulus, enhancing endothelial cell resilience through repeated controlled exposures. This preconditioning helps preserve endothelial function and mitigates harmful effects associated with methylated arginine accumulation. Together, these adaptive responses provide vascular protection during early intermittent hypoxia. However, under prolonged or severe hypoxic conditions, these protective mechanisms may fail, leading to decreased DDAH activity, elevated ADMA levels, and consequent endothelial dysfunction.^[6]^

Previous studies have shown that intermittent hypoxia and cycles of hypoxia-reoxygenation in OSAS patients can enhance endothelial nitric oxide synthase (eNOS) activity, resulting in increased nitric oxide (NO) production. In our study, we aimed to explore the balance between the L-arginine–NO pathway and L-arginine–methylated arginine metabolites (ADMA, SDMA, and L-NMMA) in newly diagnosed OSAS patients who had not yet developed complications. Specifically, we sought to identify which metabolites increase or decrease as a consequence of intermittent hypoxia.

Our primary objective was to determine whether alterations in these metabolites could serve as diagnostic biomarkers for OSAS and predict potential future complications.

## 2. Materials and methods

This study is derived from a thesis project and was approved by the Local Ethics Committee for Clinical Research at Selçuk University Faculty of Medicine (approval number: 2022/20, dated 04.01.2022). It was designed as a case-control study, conducted between January 2022 and January 2023. All participants were fully informed about the study, and written informed consent was obtained prior to enrollment.

### 2.1. Study population

#### 2.1.1. Power and sample size calculation:

A total of 122 participants were included in the study, consisting of 31 healthy controls and 91 newly diagnosed OSAS patients stratified by disease severity into mild (n = 30), moderate (n = 30), and severe (n = 31) groups. The sample size was determined based on prior research indicating significant differences in serum concentrations of methylated arginine metabolites–particularly ADMA and L-NMMA–between OSAS patients and healthy individuals. Assuming a large effect size (Cohen’s d = 0.8), a statistical power of 95%, and a 2-tailed alpha level of 0.05, a minimum of 30 participants per group was calculated to be sufficient for detecting statistically significant differences via analysis of variance (ANOVA). Consequently, the final stratification ensured adequate statistical power not only for primary comparisons but also for subgroup analyses based on OSAS severity.

#### 2.1.2. Inclusion and exclusion criteria:

The study included adult patients (≥18 years of age), both male and female, who presented to the Sleep Disorders Clinic of the Pulmonology Department at Selçuk University Faculty of Medicine and voluntarily agreed to participate. PSG recordings were analyzed to classify the patients. According to the International Classification of Sleep Disorders, Third Edition (ICSD-3), individuals included in the study had an apnea-hypopnea index (AHI) ≥ 5 accompanied by symptoms associated with OSAS, or an AHI ≥ 15 regardless of symptomatology.^[7]^

Patients with central sleep apnea or with abnormal laboratory findings–including hemoglobin (Hg), aspartate aminotransferase (AST), alanine aminotransferase (ALT), glycated hemoglobin (HbA1c), bilirubin, electrolytes, creatinine, urea, homocysteine, folic acid, and thyroid function tests–were excluded from the study. Additionally, individuals with comorbid conditions that could affect study outcomes, such as cardiovascular diseases (including essential hypertension, hypercholesterolemia, hyperhomocysteinemia, acute coronary syndromes, and congestive heart failure), diabetes mellitus, hyperthyroidism, chronic kidney disease, insulin resistance, metabolic syndrome, low serum folic acid levels, or elevated homocysteine levels, were excluded based on detailed medical history.

### 2.2. Determination of serum methylarginine levels

Blood samples were collected from eligible participants after obtaining informed consent. These samples were analyzed for ADMA, SDMA, L-NMMA, arginine levels, complete blood count, renal function tests, lipid panel, thyroid function tests, fasting blood glucose, HbA1c, folic acid, and homocysteine.

Mass spectrometric analyses were performed using an ABSCIEX API 3200 triple quadrupole mass spectrometer (USA) equipped with an electrospray ion source (ESI) operating in positive mode, integrated with a Shimadzu LC-20-AD (Kyoto, Japan) chromatography system. Chromatographic separation was achieved using a Phenomenex C18 column as the stationary phase and a gradient elution of mobile phases A and B as the mobile phase. Mobile phase A consists of a mixture of HPLC-grade water containing 0.1% formic acid, while mobile phase B consists of a mixture of methanol containing 0.1% formic acid. The percentage of mobile phase B was programmed as follows: 0.1 minute, 15%; 1.0 minutes, 25%; 2.0 minutes, 100%; 2.10 minutes, 15%; 4.90 minutes, 15%.

The ion transitions determined for each analyte were as follows (parent ion/product ion; m/z): ADMA-259.3/214; SDMA-259.3/228.0; arginine-231.3/70.0; L-NMMA-245.3/70.2; d7-ADMA-266.61/221. The ion spray voltage, source temperature, curtain, ion source (GS1), and ion source (GS2) gas values were 5000 V, 350 °C, 20, 40, and 60 psi, respectively.

The pretreatment steps applied to extract the analytes from the matrix and prepare them for analysis are summarized below.

Serum ADMA, SDMA, L-NMMA, and arginine levels were measured using the method developed by Onmaz et al (Onmaz et al, 2021). Briefly, 200 μL of serum sample was taken into Eppendorf tubes, 100 μL of internal standard (10 μm d7-ADMA) and 1000 μL of methanol were added to the mixture for protein precipitation, then vortexed for 30 seconds and centrifuged at 13,000 rpm for 10 minutes.

800 μL of the supernatant was transferred to glass tubes and evaporated under nitrogen gas at 65°C. For derivatisation, 200 μL of freshly prepared 5% (v/v) acetyl chloride in butanol solution was added. The tubes were incubated at 65°C for 30 minutes with the caps closed. After incubation, the derivatisation solution was evaporated under nitrogen gas.

The residues in the tubes were dissolved in 200 μL of a water-methanol (90:10, v/v) mixture containing 0.1% (v/v) formic acid and injected into the ABSCIEX API 3200 LC-MS/MS (MDS-Sciex®, Concord, Canada) system at 40 μL.

The intra- and inter-assay %CV values of the method were below 9.8%, and the recovery values ranged from 94.1% to 108.9%.

### 2.3. Statistical analysis

All statistical analyses were performed using R software version 3.6.0 (The R Foundation for Statistical Computing, Vienna, Austria; https://www.r-project.org). Prior to analysis, the normality of the data distribution was assessed using the Shapiro–Wilk test and Q-Q plots. The homogeneity of group variances was evaluated using Levene’s test.

Descriptive statistics for continuous variables were expressed as mean ± standard deviation or median (interquartile range), and categorical variables were presented as frequency (n). Demographic characteristics, laboratory parameters, and clinical indices of study participants across the different groups were compared using 1-way analysis of variance (ANOVA), Welch’s *F* test, or Kruskal–Wallis test, as appropriate.

For variables with statistically significant differences, post hoc pairwise comparisons were conducted using the Tukey HSD test, Games-Howell test, or Bonferroni-corrected Dunn’s test, depending on the assumptions met.

Because age distribution differed significantly among groups, age was included as a covariate in subsequent analyses. Group comparisons for laboratory findings and clinical indices were reevaluated using analysis of covariance (ANCOVA) and generalized linear models to control for the potential confounding effect of age.

A *P*-value of < .05 was considered statistically significant for all hypothesis testing.

## 3. Results

**Figure 1.**
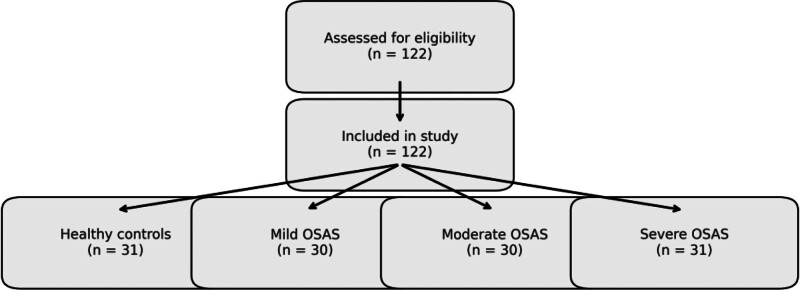
STROBE flow diagram showing the inclusion and stratification of all 122 participants in the study. All patients who applied to the PSG (polysomnography) clinic were evaluated. Screening was performed to identify patients with exclusion criteria including central sleep apnea, abnormal laboratory findings (e.g., hemoglobin, liver/kidney/thyroid function, homocysteine, folic acid), and comorbid conditions (e.g., cardiovascular disease, diabetes, hypertension, obesity). Patients without exclusion criteria were deemed eligible and included in the study. Participants were allocated to a control group (n = 31) or an OSAS group (n = 91), with the OSAS group further stratified by severity into mild (n = 30), moderate (n = 30), and severe (n = 31). OSAS = obstructive sleep apnea syndrome.

A total of 122 participants were included in the study, with 31 (25.4%) assigned to the healthy control group and 91 diagnosed with OSAS. The OSAS group was further stratified by severity into mild (n = 30, 24.6%), moderate (n = 30, 24.6%), and severe (n = 31, 25.4%) subgroups (Fig. 1).

Table 1 presents the comparison of demographic characteristics, laboratory parameters, and clinical indices between the healthy control group and OSAS patients. There was no statistically significant difference in gender distribution among the groups (*P* = .169).

**Table 1:** Comparison of demographic characteristics, laboratory parameters, and clinical indices between healthy controls and OSAS patients.

	Healthy controls (n = 31)	OSAS (n* = 91*)	*P*-value	Adjusted *P*-value (age)
Demographic characteristics				
Age (yr)	38.39 ± 9.70	45.02 ± 9.97	.002^c^	
Sex (male/female)	17/14	69/22	.047†	
Laboratory parameters				
ADMA (µM)	0.31 ± 0.05	0.19 ± 0.06	<.001‡	<.001
SDMA (µM)	0.29 ± 0.05	0.29 ± 0.06	.798*	.257
L-NMMA (µM)	0.024 ± 0.005	0.013 ± 0.006	<.001*	<.001
Arginin (µM)	89.85 ± 29.15	76.78 ± 33.14	.053*	.042
Arjinin/ADMA	286.35 (252.52–361.35)	397.22 (252.32–602.07)	.007§	.004
TMAL (µM)	0.62 ± 0.09	0.49 ± 0.10	<.001*	<.001
Clinical indices				
AHI	2.50 (1.05–3.55)	21.50 (11.10–39.70)	<.001§	<.001
Mean O_2_ saturation (%)	94.26 ± 1.48	91.03 ± 2.36	<.001*	<.001
Lowest O_2_ saturation (%)	89.48 ± 2.41	77.40 ± 8.80	<.001‡	<.001
Desaturation index	2.70 (1.40–3.80)	24.20 (13.10–43.90)	<.001‡	<.001

Values are presented as mean ± standard deviation or median (interquartile range).

ADMA = asymmetric dimethylarginine, AHI = apnea-hypopnea index, L-NMMA = N-monomethyl-L-arginine, OSAS = obstructive sleep apnea syndrome, SDMA = symmetric dimethylarginine, TMAL = total methylated arginine load.

* Independent samples *t*-test.

† Yates’ corrected Chi-square test.

‡ Welch’s *F*-test.

§ Mann–Whitney *U* test.

Table 2 shows the comparison of demographic characteristics, laboratory findings, and clinical indices across the mild, moderate, and severe OSAS subgroups. The mean age of the severe OSAS group was significantly higher than that of the mild OSAS group, while similar to that of the moderate OSAS group. The mean age of the moderate OSAS group did not differ significantly from that of the mild OSAS group, and the age distribution of the mild OSAS group was comparable to that of healthy controls.

**Table 2:** Comparison of demographic characteristics, laboratory parameters, and clinical indices across OSAS severity groups.

	Healthy controls (n = 31)	OSAS groups			*P*-value	Adjusted *P*-value (age)
		Mild (n = 30)	Moderate (n = 30)	Severe (n = 31)		
Demographic characteristics						
Age (yr)	38.39 ± 9.70^a^	40,90 ± 9,74^ac^	46,40 ± 10,25^bc^	47.68 ± 8.89^b^	<.001*	
Sex (male/female)	17/14	23/7	22/8	24/7	.169†	
Laboratory parameters						
ADMA (µM)	0.31 ± 0.05^a^	0.19 ± 0.09^b^	0.18 ± 0.05^b^	0.21 ± 0.04^b^	<.001‡	<.001
SDMA (µM)	0.29 ± 0.05	0.29 ± 0.05	0.28 ± 0.08	0.30 ± 0.05	.621*	.277
L-NMMA (µM)	0.024 ± 0.005	0.011 ± 0.006^b^	0.012 ± 0.007^b^	0.017 ± 0.004^c^	<.001‡	<.001
Arginin (µM)	89.85 ± 29.15^a^	69.05 ± 18.68^b^	98.02 ± 28.26^a^	63.72 ± 38.70^b^	<.001‡	<.001
Arjinin/ADMA	286.35 (252.52–361.35)^a^	389.90 (251.29–601.30)	589.11 (449.57–740.78)^b^	261.62 (162.40–391.98)^a^	<.001§	<.001
TMAL (µM)	0.62 ± 0.09^a^	0.49 ± 0.11^b^	0.47 ± 0.11^b^	0.52 ± 0.07^b^	<.001‡	<.001
Clinical indices						
AHI	2.44 ± 1.36^a^	9.16 ± 2.52^b^	21.81 ± 4.08^c^	54.39 ± 18.90^d^	<.001‡	<.001
Mean O_2_ Saturation (%)	94.26 ± 1.48^a^	92.20 ± 1.61^b^	90,87 ± 2,27^bc^	90.06 ± 2.63^c^	<.001*	<.001
Lowest O_2_ Saturation (%)	89.48 ± 2.41^a^	83.63 ± 5.26^b^	77.40 ± 5.72^c^	71.35 ± 9.86^d^	<.001‡	<.001
Desaturation Index	2.94 ± 2.05^a^	10.24 ± 3.88^b^	23.59 ± 4.97^c^	61.90 ± 23.07^d^	<.001‡	<.001

Values are presented as mean ± standard deviation or median (interquartile range).

Superscript letters (a, b, c, d) indicate statistically significant differences between groups (*P* < .05).

ADMA = asymmetric dimethylarginine, AHI = apnea-hypopnea index, L-NMMA = N-monomethyl-L-arginine, OSAS = obstructive sleep apnea syndrome, SDMA = symmetric dimethylarginine, TMAL = total methylated arginine load.

* Statistical test: 1-way ANOVA.

† Statistical test: Pearson Chi-square.

‡ Statistical test: Welch’s *F* test.

§ Statistical test: Kruskal–Wallis test.

### 3.1. Upon analysis of laboratory parameters

**Figure 2.**
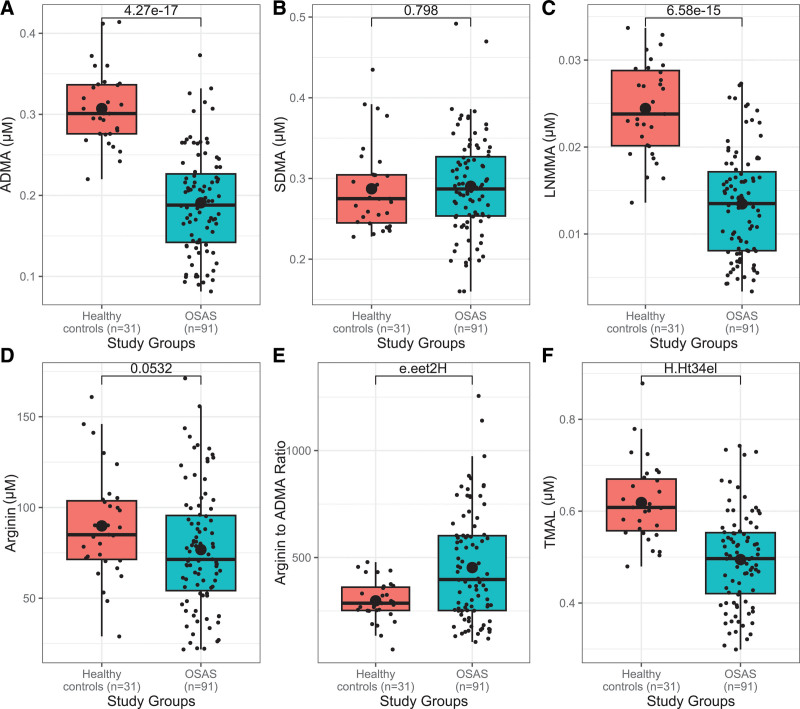
The boxplot of methylated arginine metabolites–asymmetric dimethylarginine (ADMA), symmetric dimethylarginine (SDMA), and N-monomethyl-L-arginine (L-NMMA)–and arginine, and total methylated arginine load (TMAL) parameters in study groups.

Figure 2 compares methylated arginine metabolites (ADMA, SDMA, L-NMMA), arginine, TMAL levels, and the arginine/ADMA ratio between OSAS patients and healthy controls.

ADMA levels were significantly lower in mild (0.19 ± 0.09), moderate (0.18 ± 0.05), and severe (0.21 ± 0.04) OSAS patients compared to healthy controls (0.31 ± 0.05) (*P* < .001). These results remained consistent after adjusting for age (*P* < .001).

SDMA levels did not differ significantly between OSAS patients and healthy controls, even after adjustment for age (*P* = .277).

L-NMMA levels were significantly lower in OSAS patients than in controls (*P* < .001). Notably, mild and moderate OSAS patients had lower levels than both healthy controls and those with severe OSAS (*P* < .001). Similarly, severe OSAS patients also showed significantly lower levels compared to healthy controls (*P* < .001). These trends remained unchanged after controlling for age (*P* < .001).

Arginine levels were significantly lower in patients with mild and severe OSAS compared to healthy controls and those with moderate OSAS, with age-adjusted analyses confirming the same (*P* < .001).

The arginine/ADMA ratio was significantly higher in patients with moderate OSAS compared to both healthy controls and those with severe OSAS, while it was similar between the mild OSAS and control groups (*P* < .001).

The total methylated arginine load (TMAL) was significantly lower in all OSAS groups–mild, moderate, and severe–compared to healthy controls (*P* < .001).

After adjusting for age, the following findings also remained significant:

ADMA levels were significantly lower in OSAS patients (0.19 ± 0.06) compared to healthy controls (0.31 ± 0.05) (*P* < .001).

L-NMMA levels were significantly lower in OSAS patients (0.013 ± 0.006) than in healthy controls (0.024 ± 0.005) (*P* < .001).

TMAL was significantly reduced in OSAS patients (0.49 ± 0.10) relative to healthy controls (0.62 ± 0.09) (*P* < .001).

Arginine levels were also significantly lower in OSAS patients than in healthy controls (*P* = .042).

Methylated arginine metabolites–ADMA, L-NMMA, TMAL, and arginine–were low in newly diagnosed patients who had not yet developed comorbidities, which was consistent with the clinical findings. This showed that the harmful effects of intermittent hypoxia in these OSAS patients were compensated for by other mechanisms such as NO.

### 3.2. Clinical indices

The mean oxygen saturation (O_2_) was lower in OSAS patients compared to healthy controls (*P* < .001). Among OSAS subgroups, the mean O_2_ saturation was lowest in the severe OSAS group. Patients with moderate OSAS had similar mean O_2_ levels to those with mild and severe OSAS.

The lowest oxygen saturation decreased in accordance with OSAS severity, being significantly lower in OSAS patients compared to controls and lowest in the severe OSAS group (*P* < .001).

The desaturation index (DESAT) increased with the severity of OSAS and was significantly higher in OSAS patients compared to healthy controls (*P* < .001).

## 4. Discussion

In this study, we compared plasma concentrations of methylated arginine metabolites in 91 patients with mild, moderate, or severe OSAS and 31 healthy controls. Our analysis revealed that levels of ADMA and L-NMMA were significantly lower in the OSAS group compared with controls, whereas SDMA levels remained similar across all groups. Additionally, the TMAL, defined as the sum of ADMA, SDMA, and L-NMMA, was reduced in patients with OSAS. Given that other laboratory parameters–including complete blood counts, renal and thyroid function tests, lipid profiles, fasting glucose, HbA1c, folic acid, and homocysteine–were within normal ranges, these findings suggest a compensatory mechanism in which arginine metabolism shifts toward increased nitric oxide synthesis. This adaptive response may help counteract hypoxia-induced vasoconstriction by enhancing vasodilation. Collectively, these results indicate that lower levels of arginine and its methylated derivatives could serve as potential biomarkers for the early detection of OSAS and aid in selecting patients for PSG, potentially reducing both diagnostic costs and clinical workload.

Oxidative stress refers to the condition in which the balance between oxidants and antioxidants is disturbed, leading to increased production of reactive oxygen and nitrogen species.^[8]^

The role of intermittent hypoxia in modulating oxidative stress in the pathophysiology of OSAS remains unclear.^[9]^Intermittent hypoxia, defined by repeated short periods of oxygen deficiency, is a key feature of diseases like OSAS. Early exposure to intermittent hypoxia triggers cellular adaptive responses that help protect organs and tissues.^[10]^ Intermittent hypoxia induces mild and temporary oxidative stress that activates endogenous antioxidant enzymes such as superoxide dismutase (SOD), catalase, and glutathione peroxidase. This increased antioxidant response may prevent excessive activation of protein arginine methyltransferases (PRMTs), thereby reducing the accumulation of methylated arginine derivatives.^[6]^

During the early phases of intermittent hypoxia, the enzyme dimethylarginine dimethylaminohydrolase (DDAH), which degrades ADMA, may maintain or increase its activity, accelerating ADMA breakdown and lowering its plasma and intracellular levels. This reduction in ADMA enhances nitric oxide production by endothelial nitric oxide synthase. In turn, NO exerts antioxidant effects and further stimulates DDAH activity, creating a positive feedback loop that helps sustain low ADMA concentrations.^[6]^

Early intermittent hypoxia, through its repetitive and controlled exposure, acts as a preconditioning stimulus that enhances endothelial cell resilience. This preconditioning helps maintain endothelial function and mitigates the harmful effects linked to the accumulation of methylated arginines. Together, these adaptive responses provide vascular protection during the initial stages of intermittent hypoxia. However, with prolonged or severe hypoxia, these mechanisms may become insufficient, resulting in reduced DDAH activity, increased ADMA levels, and subsequent endothelial dysfunction.^[6]^

NO is produced from L-arginine (L-Arg) and oxygen (O_2_) through the catalytic action of nitric oxide synthase (NOS). Three NOS isoforms have been identified in humans: neuronal NOS (nNOS), inducible NOS (iNOS), and endothelial NOS (eNOS). nNOS is primarily localized in neurons; iNOS is expressed in various cell types in response to proinflammatory cytokines; and eNOS is almost exclusively expressed in endothelial cells.^[11]^

The tissue-specific expression of eNOS is regulated by epigenetic mechanisms, including distinct DNA methylation patterns and post-translational histone modifications.^[12]^ Each NOS isoform generates NO at different rates, with NO concentration being a key determinant of its biological function. iNOS is the most potent NO donor, generating high concentrations of NO with cytostatic and cytotoxic effects (e.g., by activated macrophages). In contrast, eNOS produces lower NO levels that activate soluble guanylate cyclase (sGC) to generate cyclic guanosine monophosphate (cGMP), leading to vasorelaxation and inhibition of platelet aggregation, thereby preventing atherogenesis.^[13]^ Thus, endothelium-derived NO plays a critical role in maintaining vascular homeostasis, and proper eNOS activity is essential for vascular health.^[14]^

eNOS expression and activity are regulated at transcriptional, post-transcriptional, and post-translational levels. Disruptions at any point in this complex regulation can affect NO bioavailability. Various stimuli such as oxidative stress, inflammation, and hypoxia can influence eNOS expression and activity. Although hypoxia is a well-established modulator of eNOS, research findings in this area are inconsistent. The regulation of eNOS expression under hypoxia varies depending on species, endothelial heterogeneity across vascular beds, experimental models (e.g., in vitro cell culture or animal studies), and developmental stages.^[6]^

Interestingly, in bovine pulmonary artery endothelial cells, human saphenous vein endothelial cells, and in vivo in lungs of patients with pulmonary hypertension or in the aortas and mesenteric arteries of mice exposed to chronic intermittent hypoxia, the effect of hypoxia on eNOS may differ depending on whether the endothelium is arterial or venous. In contrast to human umbilical vein endothelial cells, hypoxia was shown to upregulate eNOS expression in human umbilical artery endothelial cells. Hypoxia-induced upregulation of eNOS has also been observed in the pulmonary endothelium of hypoxic rodents and in hypoxic porcine aortic endothelial cells. Chronic hypoxia similarly increased eNOS expression in the uterine endothelium of pregnant sheep.^[15]^ Some studies suggest that while hypoxia does not alter eNOS expression, it may affect enzymatic activity.^[16]^

Hypoxia may also influence the availability of L-Arg, the substrate for eNOS-mediated NO production. Intracellular L-Arg levels depend on whole-body protein turnover, endogenous synthesis, cellular uptake, and metabolism, with most plasma L-Arg derived from protein degradation.^[16]^

Intermittent hypoxia-normoxia cycles activate key transcriptional mediators such as hypoxia-inducible factor 1 (HIF-1) and nuclear factor erythroid 2 (Nrf2). These mediators upregulate cytoprotective proteins. HIF-1, a heterodimeric helix-loop-helix protein, forms a functional complex in the nucleus under hypoxic conditions and activates the transcription of target genes such as erythropoietin (EPO) and vascular endothelial growth factor.^[10]^ EPO activates protective signaling pathways and is known to reduce ischemic injury in cardiac, renal, and neural tissues. Vascular endothelial growth factor contributes to angiogenesis and neurotrophic processes by increasing vascular density and perfusion. Therefore, HIF-1 activation may significantly mitigate cerebral ischemic injury following hypoxic preconditioning.^[17,18]^

Reactive oxygen species, one of the primary initiators of adaptive responses to intermittent hypoxia, are generated during the initial phase of reoxygenation. Reactive oxygen species trigger hypoxia-induced transcription and initiate the expression of specific genes that enhance antioxidant defense against cellular stress.^[19]^

Hypoxia can be classified by duration (acute or chronic) or by nature (persistent or intermittent). For example, chronic pulmonary diseases are associated with sustained hypoxia, while OSAS is characterized by cyclic episodes of hypoxia and reoxygenation.

In our study, the patient group consisted of newly diagnosed OSAS cases without comorbidities, confirmed through detailed clinical history and laboratory evaluations. In this group, we propose that intermittent hypoxia in the early stages of disease leads to enhanced antioxidant capacity, promoting NO synthesis from L-Arg to protect organs, thereby resulting in decreased levels of methylated arginine metabolites.

In a study by Scott et al, ADMA levels were found to be higher in COPD patients compared to healthy controls, although the difference was not statistically significant. In contrast, in our study, arginine metabolites, particularly ADMA, were significantly higher in the healthy group. We attribute this discrepancy to the different hypoxia mechanisms involved–chronic and sustained in COPD versus intermittent in OSAS.^[20]^

A study by Juan Liu et al examined patients with connective tissue disease, comparing those who developed pulmonary arterial hypertension and those who did not. ADMA levels were significantly elevated in the pulmonary arterial hypertension group, implicating methylated arginine metabolites in disease-related complications.^[21]^

In a study by Barceló et al, ADMA levels tended to be higher in patients with severe OSAS, although the difference was not statistically significant. These findings suggest that elevated ADMA in OSAS may be related to obesity and metabolic disturbances.^[22]^

As highlighted in the studies above, increased arginine metabolites have been associated with oxidative stress and related complications. In our study, the absence of comorbidities was confirmed by laboratory findings and detailed clinical history. Therefore, we observed reduced levels of methylated arginine metabolites in our patient group, likely reflecting increased antioxidant capacity in the early stage of OSAS aimed at preventing organ damage.

Nural et al compared serum levels of inflammatory mediators such as C-reactive protein (CRP), tumor necrosis factor-alpha (TNF-α), and ADMA among patients with COPD, OSAS, and overlap syndrome (OVS), and also investigated changes in these markers following continuous positive airway pressure (CPAP) therapy. Blood samples were collected in the morning after polysomnography and again after regular CPAP therapy. Prior to CPAP, ADMA levels were significantly lower in OSAS compared to COPD, while CRP and TNF-α levels did not differ significantly. After CPAP, CRP levels significantly decreased in both OSAS and OVS groups, whereas TNF-α and ADMA levels showed no significant changes.^[23]^This study supports the notion that elevated arginine metabolites in OSAS may not manifest in the early stages but instead rise as complications develop over time.

### 4.1. Limitations

Our study has some limitations. Firstly, the limited sample size is one of the most significant limitations. If a larger sample had been investigated, the findings might have demonstrated greater robustness and broader applicability across diverse patient populations.

Second limitation, the serum levels of methylated arginine metabolites (ADMA, SDMA, and L-NMMA) were measured only at baseline (within 24 hours of hospital admission). However, given that these biomarkers exhibit dynamic fluctuations during disease progression, serial measurements at different time points (e.g., 1 years later, 3 years later) could have provided a more comprehensive prognostic evaluation. Tracking temporal changes in these metabolites might have revealed their association with clinical outcomes (e.g., disease severity, treatment response, or long-term complications), thereby enhancing our understanding of their pathophysiological and predictive roles in OSAS.

### 4.2. Future directions

Future studies should include prospective, controlled trials designed to monitor the progression of OSAS and its complications. Additionally, future research should focus on longitudinal monitoring of serum methylated arginine metabolites (ADMA, SDMA, and L-NMMA) levels at regular intervals. Such long-term follow-up studies in OSAS patients would provide critical insights into the role of these metabolites in disease progression, long-term complications, and patient survival.

## 5. Conclusion

In our study, serum levels of arginine metabolites–specifically ADMA and L-NMMA–were found to be significantly higher in healthy controls compared to patients with newly diagnosed OSAS. We attribute this inverse relationship to the early stage of disease in our patient population, who had not yet developed OSAS-related complications. The data suggest that, in the initial phases of the disorder, intermittent hypoxia triggers a compensatory vasodilatory response by promoting a metabolic shift in the L-arginine pathway toward NO synthesis. Consequently, the methylation pathway of arginine–responsible for the formation of ADMA and L-NMMA–remains inactive or suppressed.

These findings are consistent with experimental studies in animal models exposed to intermittent hypoxia, where increased NOS activity and elevated serum NO levels were observed. Such evidence supports the notion that the NOS pathway is more active in the early stages of intermittent hypoxia, whereas the arginine methylation pathway becomes more prominent later, as complications develop.

The literature clearly highlights the role of arginine metabolites in the pathogenesis of various systemic complications. However, our study differs in that patients with comorbidities–known to increase arginine metabolite levels–were rigorously excluded. This allowed us to focus on early metabolic changes in OSAS, demonstrating that the shift toward NO synthesis may serve a protective role prior to the onset of vascular or systemic damage.

We propose that serum levels of arginine and its methylated derivatives may serve as early diagnostic markers for OSAS. These markers could aid in the selection of patients for PSG, a costly and time-intensive diagnostic procedure, and may also help reduce the clinical burden by improving screening efficiency.

## Author contributions

**Conceptualization:** Emrah Bolca, Dilek Ergün, Recai Ergün.

**Data curation:** Emrah Bolca, Dilek Ergün, Recai Ergün.

**Formal analysis:** Emrah Bolca, Dilek Ergün, Fikret Kanat, Ali Ünlü, Duygu Eryavuz Onmaz, Muslu Kazim Körez.

**Funding acquisition:** Emrah Bolca, Dilek Ergün.

**Investigation:** Emrah Bolca, Dilek Ergün, Recai Ergün.

**Methodology:** Emrah Bolca, Dilek Ergün, Recai Ergün, Fikret Kanat, Ali Ünlü, Duygu Eryavuz Onmaz, Muslu Kazim Körez.

**Project administration:** Emrah Bolca, Dilek Ergün.

**Resources:** Emrah Bolca, Dilek Ergün.

**Software:** Fikret Kanat, Ali Ünlü, Duygu Eryavuz Onmaz, Muslu Kazim Körez.

**Supervision:** Emrah Bolca, Dilek Ergün, Recai Ergün, Fikret Kanat.

**Validation:** Ali Ünlü, Duygu Eryavuz Onmaz, Muslu Kazim Körez.

**Visualization:** Emrah Bolca, Dilek Ergün, Fikret Kanat.

**Writing – original draft:** Emrah Bolca, Dilek Ergün, Recai Ergün.

**Writing – review & editing:** Emrah Bolca, Dilek Ergün, Recai Ergün.
